# Predictors of Better Self-Care in Patients with Heart Failure after Six Months of Follow-Up Home Visits

**DOI:** 10.1155/2013/254352

**Published:** 2013-09-04

**Authors:** Melina Maria Trojahn, Karen Brasil Ruschel, Emiliane Nogueira de Souza, Cláudia Motta Mussi, Vânia Naomi Hirakata, Alexandra Nogueira Mello Lopes, Eneida Rejane Rabelo-Silva

**Affiliations:** ^1^Cardiology Division, Heart Failure Clinic, Hospital de Clinicas de Porto Alegre, Porto Alegre, RS, Brazil; ^2^Cardiology Institute of Rio Grande Sul, Cardiology Fundation, Porto Alegre, RS, Brazil; ^3^Graduate Program of Cardiovascular Science, Federal University of Rio Grande do Sul, Porto Alegre, RS, Brazil; ^4^Federal University of Health Science of Porto Alegre, Porto Alegre, RS, Brazil; ^5^Statistics Division, Hospital de Clinicas de Porto Alegre, Porto Alegre, RS, Brazil; ^6^Graduate Program, School of Nursing, Federal University of Rio Grande do Sul, Rua São Manoel 963, Bairro Rio Branco, 90620-110 Porto Alegre, RS, Brazil

## Abstract

This study aimed to examine the predictors of better self-care behavior in patients with heart failure (HF) in a home visiting program. This is a longitudinal study nested in a randomized controlled trial (ISRCTN01213862) in which the home-based educational intervention consisted of a six-month followup that included four home visits by a nurse, interspersed with four telephone calls. The self-care score was measured at baseline and at six months using the Brazilian version of the European Heart Failure Self-Care Behaviour Scale. The associations included eight variables: age, sex, schooling, having received the intervention, social support, income, comorbidities, and symptom severity. A simple linear regression model was developed using significant variables (*P* ≤ 0.20), followed by a multivariate model to determine the predictors of better self-care. One hundred eighty-eight patients completed the study. A better self-care behavior was associated with patients who received intervention (*P* < 0.001), had more years of schooling (*P* = 0.016), and had more comorbidities (*P* = 0.008). Having received the intervention (*P* < 0.001) and having a greater number of comorbidities (*P* = 0.038) were predictors of better self-care. In the multivariate regression model, being in the intervention group and having more comorbidities were a predictor of better self-care.

## 1. Introduction

Self-care in heart failure (HF) is defined as positive behaviors leading to decisions and actions that an individual can take to help maintain clinical stability and cope with the disease [1]. Studies indicate that the inability of patients to recognize signs and symptoms of congestive episodes and the lack of knowledge and poor adherence to treatment, components that are considered self-care measures, are precipitating factors leading to decompensation of HF [2–5]. Within this context, all self-care behaviors appear to be directly related to motivation, habits, and sociodemographic and clinical characteristics, factors that may affect the way individuals live their lives [1, 6–8]. 

In this sense, the home environment has gained attention as a potential setting for the development of education strategies and followup of patients with HF, as well as for the investigation of the benefits of such strategies and their effect on self-care behaviors [9–11]. Jaarsma et al. [9], in one of the first published studies on the topic, tested in a randomized controlled trial (RCT) the effect of an education strategy on self-care initiated during hospitalization, followed by a telephone call and a home visit within 10 days after hospital discharge. In the intervention group, the results demonstrated a significant improvement in self-care behavior and adherence to treatment and fewer hospital readmissions within three months of discharge [9]. Recently, two RCTs developed in Latin America have also reported improvement in self-care skills and behavior of patients receiving home visits, telephone calls, and additional written instructions [10, 11].

Although recent studies have shown favorable results in patients receiving home visits for self-care education, in Latin America the predictors of a better self-care behavior in patients with HF are yet to be explored in this setting. This study aimed to examine the predictors of better self-care behavior in patients with HF in a home visiting program.

## 2. Patients and Methods

### 2.1. Study Design

This is a longitudinal study nested in an RCT called HELEN-I (ISRCTN01213862), whose primary aim was to verify the effect of a nursing educational intervention consisting of home visits and phone calls on patients' knowledge of the disease, self-care, and adherence to treatment. The results showed that alternating home visits with telephone calls during a six-month follow-up period for patients hospitalized for decompensated HF improved patients' knowledge of the disease, self-care skills, and adherence to treatment [11].

### 2.2. Participants

The sample included 188 adult patients diagnosed with HF and systolic dysfunction, hospitalized for decompensated HF. Patients with communication barriers, with a diagnosis of acute HF secondary to sepsis, myocarditis, or acute myocardial infarction, who lived more than 20 km from the institutions, or who could not be contacted by telephone were excluded from the study. Hospitalized patients were recruited from inpatient medical units and emergency departments by active search during daily visits by the study team to these units. The study was conducted at two referral centers for the treatment of patients with HF in the metropolitan area of Porto Alegre, city capital of Rio Grande do Sul, the southernmost state of Brazil, and approved by the Research Ethics Committees of both institutions (protocol numbers 09-111 and 4339-09).

### 2.3. Data Collection and Study Intervention

Data on sociodemographic and clinical characteristics of patients were collected at baseline (upon inclusion in the study) and six months after hospital discharge. Eight sociodemographic and clinical variables were chosen to be tested for association with the outcome (better self-care): age, sex, years of schooling, having received the home-based educational intervention, social support, family income, comorbidities, and symptom severity.

The intervention group received four home visits by a nurse, interspersed with four telephone calls, in a six-month follow-up period for education on the disease, adherence to treatment, and self-care practices. The control group received all standard care in their institutions of origin, with no home visits or telephone contact. Both groups were assessed after six months of followup in the referral hospitals [11]. 

Self-care was assessed using the Brazilian version of the European Heart Failure Self-Care Behaviour Scale (EHFScBS) [12]. It consists of 12 questions that cover items concerning daily self-care activities: daily weighing (item 1), symptom recognition and seeking assistance (items 2–5), fluid restriction (item 6), daily rest (item 7), recognition of symptom worsening and seeking assistance (item 8), sodium restriction (item 9), correct use of medications (item 10), annual influenza vaccination (item 11), and exercising regularly (item 12). Scores are divided according to the patient's response on self-care practices. Each item is rated on a five-point scale between 1 (I completely agree) and 5 (I completely disagree), and lower scores indicate a better self-care behavior. Individual item scores are summed up to give a total score, ranging from 12 to 60.

### 2.4. Statistical Analysis

Data were analyzed using the Statistical Package for the Social Sciences (SPSS) version 18.0. Continuous variables were expressed as mean and standard deviation if normally distributed, and as median and interquartile range (25th–75th percentiles) if not normally distributed. Categorical variables were expressed as count and percentage. Relationships between sociodemographic and clinical variables and the self-care score were examined using a simple linear regression model. Variables that reached *P* ≤ 0.20 in the univariate analysis were subsequently included in the multivariate analysis (multiple regression) in order to determine the predictors of better self-care. Comparisons between groups were performed using Student's *t* test for continuous variables and the self-care score. Values were considered to be statistically significant if *P* value was <0.05.

## 3. Results

### 3.1. Sociodemographic and Clinical Characteristics

A total of 188 patients were included in the study, 91 in the intervention group and 97 in the control group. The sociodemographic and clinical characteristics of patients are described in Table 1. Ischemic heart disease was the most common etiology of HF, and most patients were in New York Heart Association (NYHA) functional class III. The variables were not significantly different between groups.

### 3.2. Association between Sociodemographic and Clinical Characteristics and Self-Care Score at the End of Six Months

Eight sociodemographic and clinical variables (age, sex, years of schooling, having received the intervention, social support, family income, comorbidities, and symptom severity) were tested for association with a better self-care behavior at the end of six months of followup. Better self-care was associated with patients who received the educational intervention (*P* < 0.001), had more years of schooling (*P* = 0.016), and had more comorbidities (*P* = 0.008) (Table 2).

### 3.3. Predictors of Self-Care at the End of Six Months

In the model that included six variables with *P* ≤ 0.20 (sex, years of schooling, having received the intervention, social support, family income, and comorbidities), having received the educational intervention (*P* < 0.001) and having a greater number of comorbidities (*P* = 0.038) were predictors of better self-care (Table 3).

## 4. Discussion

This study is the first in Latin America to examine the association between sociodemographic and clinical variables and predictors of self-care in patients with HF. Patients who received home-based educational intervention, who had more years of schooling and who had a greater number of comorbidities showed a strong association with a better self-care behavior. Regarding the predictors, having received the educational intervention and having more comorbidities were identified as predictors of better self-care.

Previous studies have reported that patients with more than six years of schooling have a better self-care behavior due to better understanding of and consequent improved adherence to treatment [6, 8]. In a study involving 209 patients admitted to six hospitals in California, United States, schooling was identified as a predictor of better treatment adherence and better self-care (*P* = 0.009). Those authors demonstrated that formal education is generally associated with higher income levels, thus facilitating self-care [8]—although income was not a significant predictor in the present study. Studies suggest that formal education is associated with better understanding of and consequent greater adherence to recommendations and treatment, aspects that help patients remain clinically stable [8, 13].

In the present study, having a greater number of comorbidities was a predictor of self-care, which is not consistent with the findings from a study involving inpatients from two hospitals in the United States [2]. However, other authors have shown that people with multiple chronic diseases are more likely to monitor their health in order to avoid clinical instability and subsequent hospitalization [14]. More recent studies contradict this hypothesis, suggesting that patients with multiple comorbidities find it more difficult to recognize the symptoms of each disease, which hinders their ability to learn about self-care and to recognize specific signs and symptoms [2, 15]. Likewise, poor adherence to drug therapy, especially because of the large number of drugs taken, may be a potential barrier to the successful self-management of HF [16]. 

Studies conducted to evaluate self-care behaviors among patients with HF suggest that having more years of schooling, more symptoms, fewer comorbidities, and being male are the most common predictors of self-care [2, 8]. In this study, having received the educational intervention and having more comorbidities were the only variables identified as predictors of self-care. These results suggest that educational approaches to improve patients' knowledge of the disease and treatment should be implemented in different settings and that sociodemographic and clinical characteristics should be considered by the health care team in order to point out those variables predicting a better or worse self-care behavior.

Patient compliance with treatment regimens, aiming to achieve clinical stability in the followup of patients with HF, is a major challenge for health care professionals, and in this context self-care is a key element. For patients with HF, positive practices, such as weight control, fluid and sodium restriction, physical activity, annual vaccination, regular use of medication, and especially the development of skills for early recognition of signs and symptoms of decompensated HF and decision making when symptoms occur, are beneficial behaviors to achieve and maintain clinical stability over the long term.

## 5. Conclusions

Based on the present results, we can conclude that there was an association between a better self-care behavior and being allocated to the intervention group (who received education on the disease, self-care practices, and treatment), having more years of schooling and more comorbidities. In addition, being in the home visiting program and having a greater number of comorbidities were a predictor of better self-care. This study becomes relevant once sociodemographic and clinical predictors have been identified that lead to a better self-care behavior in patients with HF.

These results also highlight the importance of examining aspects associated with better self-care practices, as well as behaviors and education strategies to guide the patient toward better self-care skills. The recognition of these variables by the multidisciplinary health care team may help guide decisions about the best approach for patient followup.

## Figures and Tables

**Table 1:** Sociodemographic and clinical characteristics (*n* = 188).

Characteristics	Intervention group *n* (%)	Control group *n* (%)	*P*
Patients	91 (48.4)	97 (51.5)	
Age, years*	62.9 ± 13.5	62.9 ± 13	0.964
Caucasian	62 (68.1)	64 (66.0)	0.922
Sex, male	54 (59.3)	61 (62.9)	0.618
Marital status, married	58 (63.7)	60 (61.9)	0.530
Social support, family	82 (90.1)	82 (85.4)	0.330
Schooling, years*	6.81 ± 4.33	6.16 ± 4.28	0.317
Income, total value in US dollars^†^	600 (450–900)	650 (387–900)	0.698
Duration of disease, years^†^	5 (1.75–12.7)	6 (3.5–11.5)	0.216
Etiology, ischemic	25 (35.4)	27 (32.9)	0.166
NYHA functional class^‡^			0.932
I	6 (6.9)	9 (9.4)	
II	35 (40.2)	36 (37.5)	
III	40 (46)	44 (45.8)	
IV	6 (6.9)	7 (7.3)	
Ejection fraction*	29.9 ± 10.8	31.4 ± 13.9	0.421
Comorbidities			
Systemic hypertension	56 (61.5)	70 (72.2)	0.121
Diabetes mellitus	35 (38.5)	34 (35.1)	0.628
Acute coronary syndrome	25 (27.5)	35 (36.1)	0.206

Categorical variables are expressed as *n* (%), and continuous variables are expressed as *mean ± standard deviation or ^†^median and 25th–75th percentiles; ^‡^New York Heart Association functional class; information was missing for 4 cases in the intervention group and 1 case in the control group.

**Table 2:** Association between sociodemographic and clinical characteristics and the self-care score at the end of six months.

Variables	*P*
Age	0.400
Sex	0.101
Years of schooling	0.016
Having received the intervention	0.001
Social support	0.197
Comorbidities	0.008
Symptom severity	0.110
Income	0.073

**Table 3:** Predictors of self-care at the end of six months in the model with six variables (*n* = 188).

Predictor variables	*B*	SE	*β*	*t*	*P*
Intervention group	−7.51	0.97	−0.49	−7.73	0.000
Sex	−1.34	1.04	−0.08	−1.28	0.200
Years of schooling	−1.10	0.125	−0.59	−0.85	0.392
Comorbidities	0.66	0.308	0.14	2.16	0.038
Social support	1.35	1.52	0.58	0.852	0.373
Income	−0.00	0.001	−0.03	−0.48	0.626

*R* ^2^ = 0.336—Coefficient of determination (general model).
